# Commentary: Clinical Improvements in Comorbid Gambling/Cocaine Use Disorder (GD/CUD) Patients Undergoing Repetitive Transcranial Magnetic Stimulation (rTMS)

**DOI:** 10.3389/fncir.2020.00039

**Published:** 2020-07-24

**Authors:** Hang Ou, Yi Zhang, Weiqi He

**Affiliations:** ^1^Research Center of Brain and Cognitive Neuroscience, Liaoning Normal University, Dalian, China; ^2^Key Laboratory of Brain and Cognitive Neuroscience, Liaoning Province, Shanghai, China; ^3^Shanghai Mental Health Center, School of Medicine, Shanghai Jiao Tong University, Shanghai, China

**Keywords:** transcranial magnetic stimulation, substance use disorder, gambling disorder, comorbid gambling/cocaine use disorder, clinical improvement, neuromodulation

Addiction is a multidimensional psychiatric disorder that can involve various addictive agents (drug, gambling, sex, shopping, games, etc.) (Lee et al., 2019). In recent years, a subtype of non-substance behavioral addiction, gambling disorder (GD), has been receiving increased attention. Reclassified from the category of impulse-control disorders, GD is now a new category labeled “Substance-Related and Addictive Disorders” and is the only behavioral addiction recognized in the fifth edition of the Diagnostic and Statistical Manual [DSM-5, American Psychiatric Association (APA), 2013]. Notably, It is estimated that ~14% of patients with substance use disorder (SUD) have GD as well (Cowlishaw et al., 2014), since the two share many common factors, such as clinical characteristics, social factors, personality traits (Petry et al., 2005), biochemistry (Potenza, 2006), genetics (Leeman and Potenza, 2013), and neurocircuitry (van Holst et al., 2010), leading to concurrent conditions in clinical population (Rush et al., 2008; Lorains et al., 2011).

Few studies have examined the treatment options for comorbid GD/SUD. However, repetitive transcranial magnetic stimulation (rTMS) is a promising tool in SUD that works by modulating the impaired neural circuitry with prefrontal stimulation, to decrease craving or drug intake (Gorelick et al., 2014; Rapinesi et al., 2016; Shen et al., 2016; Terraneo et al., 2016; Liang et al., 2018; Lin et al., 2019; Liu et al., 2019). Recently, a few pilot studies have shown that rTMS might be an effective treatment for GD patients as well (Rosenberg et al., 2013; Zack, 2016; Gay et al., 2017).

In a recent study, Cardullo et al. (2019) investigated the effects of rTMS on cocaine-dependent individuals with GD comorbidity for the first time, providing unprecedented insights into the potential effect of rTMS as a therapeutic intervention for reducing both gambling and cocaine use in these patients. In this study, seven male participants who were diagnosed as having cocaine use disorder (CUD) and GD received the rTMS treatment over 18 sessions, with 15 Hz rTMS (2,400 pulses/session, total 43,200 pulses) over the left dorsal lateral prefrontal cortex (DLPFC). In addition, their level of gambling severity and cocaine craving, as well as sleep quality and other negative symptoms, was assessed using the Gambling-Symptom Assessment Scale (G-SAS), Cocaine Craving Questionnaire (CCQ), Pittsburgh Sleep Quality Index (PSQI), Beck Depression Inventory (BDI), Self-rating Anxiety Scale (SAS), and Symptoms checklist-90 (SCL-90), respectively. Assessments were made at baseline, immediately after completion of the first week of treatment (twice/day), as well as at 30 and 60 days (one time/week) after the first session. During the whole process, no aversive side effects were reported. Five days after the first treatment, their level of both cocaine craving and gambling severity dropped dramatically and stayed stable over time. Moreover, negative symptoms including sleep disturbance, depression, and anxiety significantly decreased compared with the baseline as reflected in their PSQI, BDI, and SAS scores. At the 60-days follow-up, four out of seven patients had not relapsed, for either cocaine use or gambling.

The treatment effects observed for GD/CUD patients were in line with previous results for patients with CUD or GD, separately. It is possible that these treatment effects were due to increased dopaminergic system activity following brain stimulation, as dopamine is implicated in both substance use and gambling. Substance-related disorder is assumed to be associated with a dysregulation of dopamine transmission resulting from learned drug-related reinforcers (Di Chiara and Imperato, 1988; Volkow et al., 2007, 2017). Similarly, the uncertainty of rewards, a core characteristic of gambling, may play the same role as drug-related reinforcers in dopamine pathways (Fiorillo et al., 2003; Zald et al., 2004). Furthermore, some studies have indicated the reciprocal priming effects of substance use and gambling. For instance, one study indicated an acute effect of a psychostimulant in individuals with pathological gambling (PG, a former name of GD), whose motivation for gambling was primed by amphetamine (Zack and Poulos, 2004). Similarly, exposure to conditions of uncertain reward also promotes the pursuit of amphetamine in rats (Mascia et al., 2019). It will be important to investigate the modification of these reward-related behavioral changes in GD/CUD patients after brain stimulation treatments.

Few studies have compared individuals who have substance dependence and pathological gambling (SDPG) with individuals who only have substance dependents (SD), in personality (Petry, 2001), cognitive function (Krmpotich et al., 2015), and brain alterations (Yip et al., 2018). According to these studies, individuals with SDPG have elevated impulsivity personality trait than those with SD when assessed by the Barratt Impulsiveness Scale (BIS-11) (Krmpotich et al., 2015), Stanford Time Perception Inventory (STPI), and Eysenck and Barratt scales (Petry, 2001). It would be interesting to know whether rTMS treatment could restore the different behavioral aspects to similar extent in SDPG and SD patients. This would provide additional support for rTMS to be used as a treatment option for comorbidity patients. A recent study highlighted the potential of prefrontal rTMS in reducing behavioral impulsivity of methamphetamine dependents (Yuan et al., 2020), which would be an implication for treating GD/SUD patients, as impulsivity is a shared feature.

This clinical research seems encouraging, as the first step toward investigating rTMS intervention in patients with SDPG. The promising effects thus far suggest that rTMS could be beneficial to comorbid cases, and may even pronounce greater efficiency than when employed with addicts with a single diagnosis. Nevertheless, considering the heterogeneity of addicts and types of addictions, patients with comorbid CUD and GD present only one subtype of addiction. Therefore, it should be careful to generalize such results of Cardullo's article to other types of addictions. In order to achieve the maximal effectiveness of rTMS as a treatment for addiction and to better understand the essence of addiction, further investigations are needed to explore other drug abuses concurrent with GD, and compare the treatment effects of rTMS in patients with SDPG and SD.

## Author Contributions

HO, YZ, and WH conceived the idea and wrote the manuscript together. All authors contributed to the article and approved the submitted version.

## Conflict of Interest

The authors declare that the research was conducted in the absence of any commercial or financial relationships that could be construed as a potential conflict of interest.
